# Using Background Sequencing Data to Anticipate DENV-1 Circulation in the Lao PDR

**DOI:** 10.3390/microorganisms9112263

**Published:** 2021-10-30

**Authors:** Elodie Calvez, Phaithong Bounmany, Charlotte Balière, Somphavanh Somlor, Souksakhone Viengphouthong, Thonglakhone Xaybounsou, Sitsana Keosenhom, Kitphithak Fangkham, Paul T. Brey, Valérie Caro, Vincent Lacoste, Marc Grandadam

**Affiliations:** 1 Arbovirus and Emerging Viral Diseases Laboratory, Institut Pasteur du Laos, Vientiane 01030, Laos; p.bounmany@pasteur.la (P.B.); s.somlor@pasteur.la (S.S.); s.viengphouthong@pasteur.la (S.V.); t.xaybounsou@pasteur.la (T.X.); s.keosenhom@pasteur.la (S.K.); k.fangkham@pasteur.la (K.F.); v.lacoste@pasteur.la (V.L.); marc.grandadam@orange.fr (M.G.); 2 Environment and Infectious Risks Unit, Institut Pasteur, 75015 Paris, France; charlotte.baliere@pasteur.fr (C.B.); valerie.caro@pasteur.fr (V.C.); 3 Lao Army Institute for Preventive Medicine, Vientiane 01030, Laos; 4 Medical Entomology and Vector Borne Disease Unit, Institut Pasteur du Laos, Vientiane 01030, Laos; p.brey@pasteur.la

**Keywords:** dengue, DENV-1, epidemiology, phylogeny, Lao PDR

## Abstract

Since its first detection in 1979, dengue fever has been considered a major public health issue in the Lao People’s Democratic Republic (PDR). Dengue virus (DENV) serotype 1 was the cause of an epidemic in 2010–2011. Between 2012 and 2020, major outbreaks due successively to DENV-3, DENV-4 and recently DENV-2 have been recorded. However, DENV-1 still co-circulated in the country over this period. Here, we summarize epidemiological and molecular data of DENV-1 between 2016 and 2020 in the Lao PDR. Our data highlight the continuous circulation of DENV-1 in the country at levels ranging from 16% to 22% among serotyping tests. In addition, the phylogenetic analysis has revealed the circulation of DENV-1 genotype I at least since 2008 with a co-circulation of different clusters. Sequence data support independent DENV-1 introductions in the Lao PDR correlated with an active circulation of this serotype at the regional level in Southeast Asia. The maintenance of DENV-1 circulation over the last ten years supports a low level of immunity against this serotype within the Lao population. Thereby, the risk of a DENV-1 epidemic cannot be ruled out in the future, and this emphasizes the importance of maintaining an integrated surveillance approach to prevent major outbreaks.

## 1. Introduction

Dengue viruses (DENVs) are the most prevalent human arboviruses worldwide as a direct consequence of the distribution of the main dengue viruses’ vector *Aedes* species (*Aedes aegypti*, *Aedes albopictus*) [1]. In 2019, 4.2 million dengue cases were reported by the World Health Organization (WHO), with 70% of the burden in Asia. The global surge in dengue between 2000 and 2015 was accompanied by more than a 4-fold increase in the number of reported deaths [2].

DENV are positive-sense RNA viruses (*Flavivirus* genus; *Flaviviridae* family) of which four distinct serotypes were identified (DENV-1 to DENV-4) [3,4,5,6]. DENV-1 was first detected in 1943 in French Polynesia and Japan and seemed to be the most reported serotype between 1943 and 2013 [7,8]. DENV-1 is subdivided into genotypes often linked to the geographical origin of the viral strains [8,9]. Five DENV-1 genotypes are currently described: genotype I (Asia), II (Thailand), III (Malaysia), IV (South Pacific) and V (America/Africa) [10,11].

The first recorded DENV outbreak in the Lao PDR occurred in 1979. Since then, the disease has been recognized as a major public health issue in the country [12,13,14]. Between 2006 and 2010, DENV-1 circulated at a high level in the Lao PDR. Over this period, this serotype accounted for 58.7% to 85.7% of the serotyped samples in Vientiane Capital and 55.9% and 80.7% of the serotyped samples in Luangnamtha (in the north) and Saravane (in the south) provinces, respectively [15,16]. Then, an outbreak of DENV-3 was reported in 2012–2013 [17]. While recent studies on DENV-4 and DENV-2 have demonstrated the low level of circulation of these two serotypes before 2014, major outbreaks were subsequently reported in 2016–2018 (DENV-4) and 2019–2020 (DENV-2), during which changes in genotypes and/or clusters within these serotypes could be observed [18,19]. In the meantime, DENV-1 continuously circulated, representing between 16% and 22% of the serotyped samples at the national level yearly [13,15,16,17,18,19]. Therefore, in the context of co-circulation of different DENV serotypes, we aimed to better characterize the circulation of DENV-1 in the Lao PDR between 2016 and 2020 through epidemiological and phylogenetic investigations.

## 2. Materials and Methods

### 2.1. Ethics Statement

The samples included in this study were collected for diagnostic purposes. The participant or a parent or legal guardian provided written informed consent for the further use of the leftovers of their samples for research purposes. Ethical approval was obtained from the Lao National Ethics Committee for Health Research of the Ministry of Health (N°2018.116).

### 2.2. Sample Collection

Blood samples (5 mL of venous blood) were collected by clinicians from the arbovirus surveillance hospital network in Vientiane Capital and some of the Lao provinces [17,18,19]. Briefly, suspected patients, presenting dengue fever symptoms matching with the WHO’s case definition (fever onset ≥38 °C for less than 7 days with at least one of the following accompanying symptoms: headache, myalgia, arthralgia, retro-orbital pain, digestive troubles or hemorrhaging), were included in this study after obtaining informed consent. Samples were stored at 4 °C during transportation to the Institut Pasteur du Laos (IPL) where serological and molecular investigations were performed.

### 2.3. Dengue Suspected Cases Screening and Serotype Identification

RNA was extracted from plasmas using purification kits (NucleoSpin Dx or NucleoSpin 96 Core Kit, Macherey-Nagel, Düren, Germany) following the manufacturer’s instructions. Samples were first screened with a pan-dengue real-time RT-PCR [20], and a specific real-time RT-PCR was used as previously described to determine the serotype [21].

### 2.4. Sequencing Analysis of the Envelope Gene

*Envelope* (E) gene sequencing (1485 nt) was performed on a panel of samples, according to their geographical origin, the year of collection, the Ct value for the pan-dengue real-time RT-PCR and the remaining sample volume, using primer sets D1-FG1, D1-FGT2 and D1-FGT3 (Table 1). The PCR products were purified with ExoSAP-IT PCR Product Cleanup Reagent (Thermo Fisher Scientific, Waltham, MA, USA) and sequenced using a BigDye Terminator v3.1 Cycle Sequencing Kit (Applied Biosystems, Waltham, MA, USA) on a 3500xL Genetic Analyzer apparatus (Applied Biosystems, Waltham, MA, USA).

The raw sequences were analyzed and edited using Chromas software (www.technelysium.com.au). A multiple-sequence alignment was constructed, with other previously published representative DENV-1 sequences downloaded from GenBank (www.ncbi.nlm.nih.gov/nucleotide/), using the ClustalW program integrated in BioEdit version 7.0.5.3 software (Manchester, UK) [15,16,22,23]. The alignment was checked manually. For phylogenetic analysis, a maximum likelihood tree was constructed using MEGA version 7 (www.megasoftware.net), with a Kimura 2-parameter model with a bootstrap of 1000 replications [24]. New clusters were designated according to the nucleotide identity (>99%) and associated with the bootstrap value (>90) [15], for a group of sequences which contains at least one sequence from the Lao PDR.

## 3. Results

### 3.1. Dengue Virus Circulation and Outbreaks in the Lao PDR

**Figure 1:**
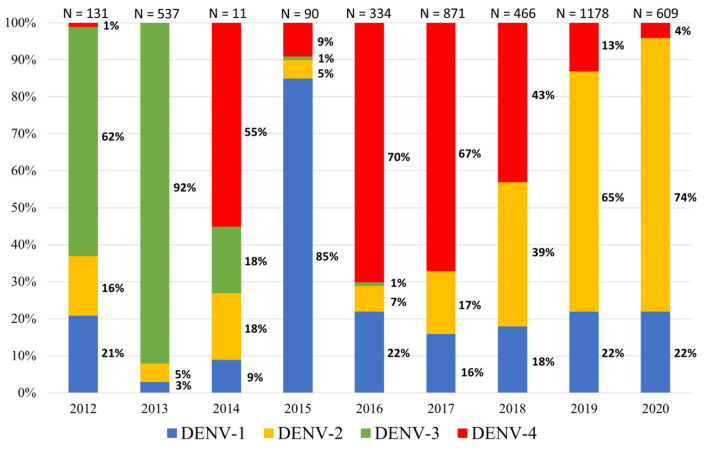
Dengue virus (DENV) serotype ratios in the Lao PDR based on samples collected by the Institut Pasteur du Laos arbovirus surveillance network between 2012 and 2020.

Between 2012 and 2019, more than 4200 DENV-positive samples were detected in the Lao provinces (Table 2). During this period, the dengue surveillance network was gradually extended. Indeed, between 2012 and 2015, the surveillance focused on Vientiane Capital and only a few samples from the other provinces could be analyzed. Since 2015, two additional provinces, Saravane and Attapeu, sent samples on a weekly basis for analysis. Epidemiological studies in the Lao PDR revealed a cascade of epidemics due to a rapid switch in the predominance of DENV serotypes (Figure 1). During this period, a successive predominance of DENV-3, DENV-4 and DENV-2 was observed in the Lao PDR, as previously described [18]. No DENV outbreak has been described in 2014–2015. Even if three of the four DENV serotypes were detected, the number of suspected cases constantly remains below the alert threshold defined by the average of the weekly cases reported during the previous five years plus two standard deviations. A total of 350 suspected cases were investigated over this period (2014–2015), within which only 101 could be confirmed for DENV infection.

Between 2012 and 2016, DENV-1 rates ranged from 3% (in 2013; N = 537) to 85% (in 2015; N = 90) in the Lao PDR (Figure 1) [18]. Since 2016, DENV-1 has only accounted for 16% to 22% of the samples serotyped by the arbovirus surveillance network (Figure 1). Interestingly, during this period DENV-1 circulation was continuous and nearly stable in contrast with the other serotypes (Figure 1).

**Figure 2:**
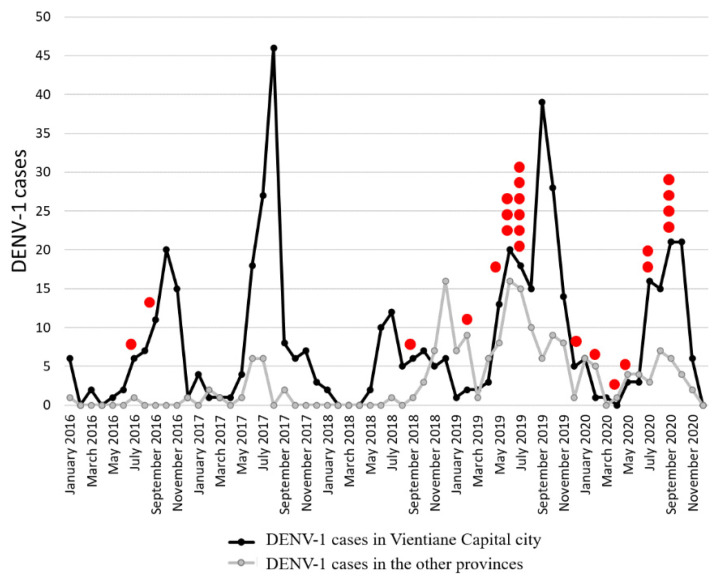
Monthly record of dengue virus serotype 1 cases in Vientiane Capital and in other provinces from the samples collected by the Institut Pasteur du Laos arbovirus network between 2016 and 2020. Samples sequenced in this study are identified by red dots.

**Figure 3:**
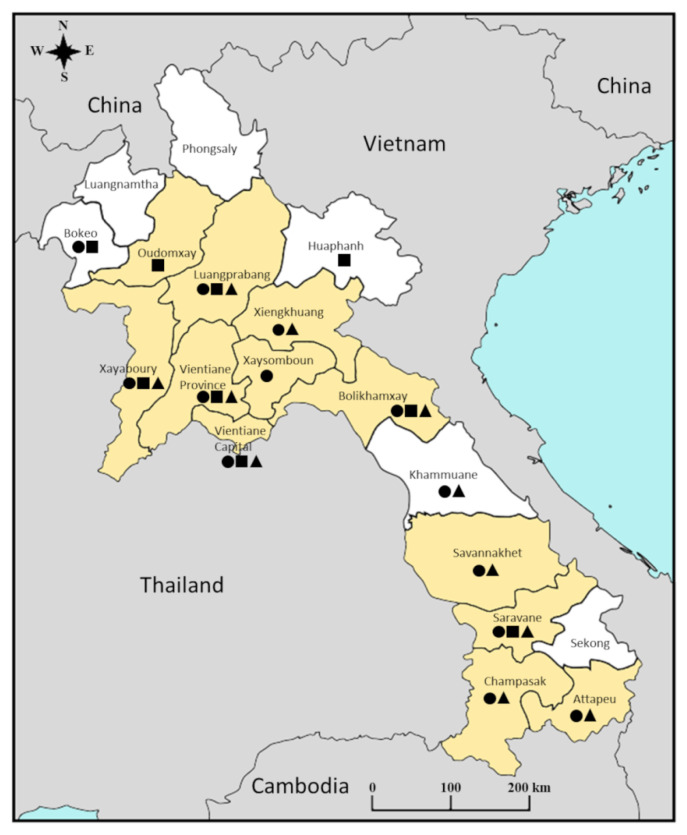
Dengue virus serotype 1 circulation in Lao provinces based on the analysis of the samples collected by the Institut Pasteur du Laos arbovirus surveillance network between 2012 and 2020. The provinces indicated in yellow correspond to those where at least one sample was found positive for dengue virus serotype 1. The dots, squares and triangles indicate provinces where at least one sample was found positive for dengue virus serotype 2, 3 or 4, respectively.

Among the samples tested, 76% of the DENV serotyped samples were from Vientiane Capital (Table 2). Between 2016 and 2020, five DENV-1 case peaks (>10 cases per month) were observed in the capital city (Figure 2). In addition, the increase in DENV-1 cases was usually concomitantly observed in the capital and in other provinces under surveillance. By contrast, in 2018, the highest number of DENV-1 cases was recorded in May in Vientiane Capital and only in November in the provinces. At the national scale, DENV-1 was recorded in 12 of the 18 Lao provinces during the study period (Figure 3). The number of DENV-1 cases usually peaked between July and November during and after the rainy season (Figure 2).

In 2020, the proportions of DENV serotypes did not change, and DENV-2 remained predominant, representing 74% of the samples collected at the national level, followed by DENV-1 (22%) and DENV-4 (4%) (Figure 1).

### 3.2. DENV-1 Phylogeny

**Figure 4:**
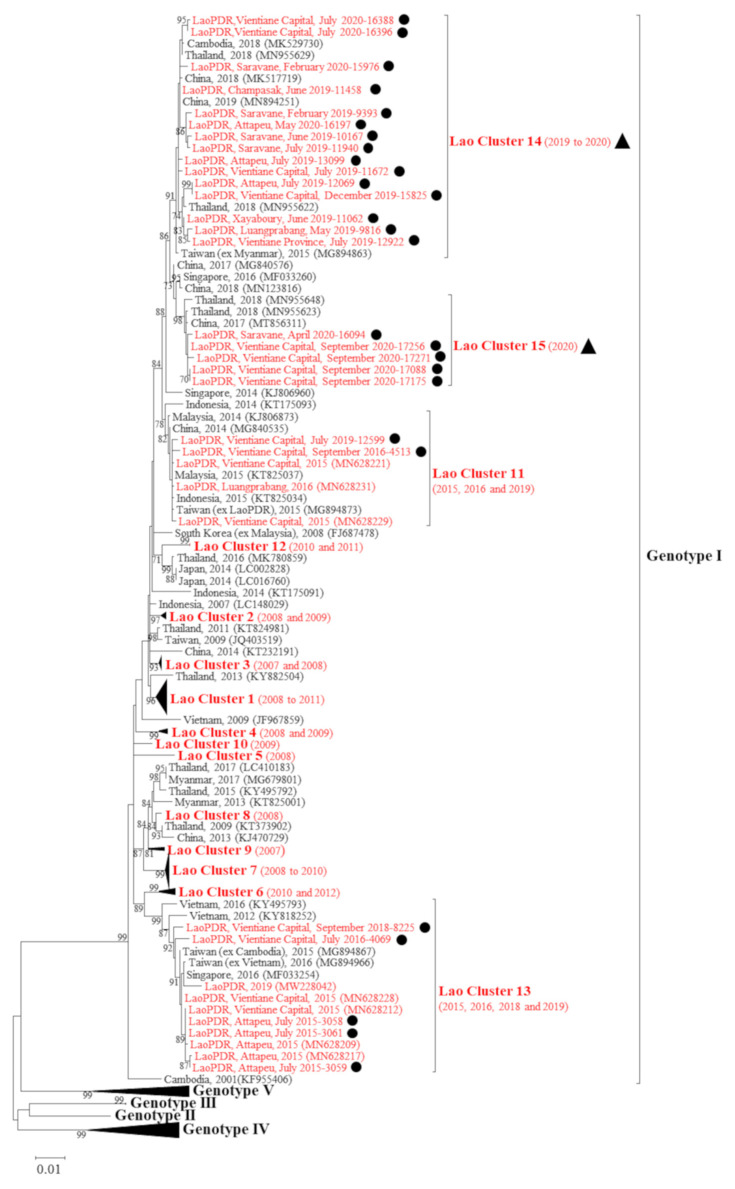
Maximum likelihood phylogenetic tree of DENV-1 sequences from the Lao PDR. The tree was constructed on the *envelope* gene (1485 nt). Only the bootstrap values > 70 are shown. Scale bar indicates the nucleotide substitution per site. The Lao strains are indicated in red. The sequences obtained in this study and the three sequences obtained in 2015 are indicated by a dot. The new clusters are marked by a triangle. For each cluster, the year of sample collection was indicated in the parentheses.

A panel of 24 DENV-1 plasma samples, collected between 2016 and 2020 in several Lao PDR provinces, were investigated for the *envelope* gene for phylogenetic analysis (Table 3). Three samples, collected in 2015 and previously sequenced, were added for phylogenetic analysis (Table 3). A fragment of 1485 nt was obtained for each sample. The phylogenetic analysis demonstrated that the new sequences all belonged to the genotype I of DENV-1 (Figure 4).

Sequences from Lao DENV-1 isolates were distributed in four distinct clusters (Figure 4). Two sequences from samples isolated in Vientiane Capital grouped in cluster 11, a cluster that had previously been observed in the Lao PDR, and shared more than 99.5% of their nucleotide identity with the former strains (C. Balière, personal communication). These sequences were closely related to sequences from Malaysia, China and Indonesia identified in 2014–2015 (>99.5% shared nucleotide identity). Five other sequences from samples collected in Attapeu Province in 2015 and in Vientiane Capital in 2016 and 2018, respectively, grouped in cluster 13. This cluster was previously observed in the Lao PDR from samples collected in the same region in 2015 (C. Balière, personal communication). All these strains shared 99% of their nucleotide identity. In addition, they clustered with sequences identified in Taiwan (imported from Cambodia and Vietnam), and in Singapore in 2015–2016 (Figure 4).

Sequences of the last 20 isolates grouped in two other new clusters: clusters 14 and 15 (Figure 4). In cluster 14, the sequences identified from samples from Vientiane Capital and Vientiane Province, as well as from Xayaboury and Luangprabang provinces to the north and from Champasak, Saravane and Attapeu provinces to the south grouped with sequences from China, Thailand and Taiwan (imported from Myanmar) dated from 2018–2019 (99.1% shared nucleotide identity). In cluster 15, the sequences from samples collected in Vientiane Capital and Saravane Province in 2020 displayed a link with sequences from China and Thailand collected in 2017 and 2018, respectively (99.3% shared nucleotide identity).

Since 2008, 15 clusters of DENV-1/genotype I have been identified in the Lao PDR. Among them, some clusters lasting no more than one year (clusters 5, 8, 7 and 10) were detected while some others lasted for longer periods with a maximum of four years (clusters 1, 2, 3, 4, 6, 7 11, 12, 13 and 14). The last cluster documented to date, cluster 15, was detected in April 2020 and its circulation was still on going at the end of the study period in September 2020. Of note, the circulation of clusters 14 and 15 was identified during the dry seasons when the number of suspected and confirmed cases are below their respective alert thresholds. All together these results documented the rapid and constant turnover of DENV-1/genotype I clusters.

## 4. Discussion

Since 1979, dengue circulation in the Lao PDR has been complex and dynamic [12,13,16,18]. The active circulation of DENV is promoted by the geographical context of the country. Indeed, the Lao PDR is located in the middle of the Indochinese Peninsula, a region characterized as hyperendemic for DENV for decades [7]. In addition, demographic factors could influence the occurrence of DENV outbreaks. The Lao population is young, with half under the age of 24 [25]. Thus, it can be assumed that an important part of the population is immunologically naïve to DENV, especially knowing that the DENV serotype barely circulated in the country for years, if at all. For instance, in 2006, in Vientiane Capital 84.6% of the adults above 35 years (N = 1990) displayed IgG reactive to DENV antigen but only 9.4% of the children (≥6 months and <6 years; N = 1568) were found to be positive [26]. Since 2012, three major outbreaks have occurred in the Lao PDR predominated by DENV-3 (2012–13), DENV-4 (2016–18) and DENV-2 (2019–20) [17,18,19]. Even if DENV-1 was detected before 2010, among the samples tested in the Lao PDR [13,15,16], its circulation over the last 10 years was not actually very widespread in a national context. The main bias in health surveillance systems in the Lao PDR is the centralization of laboratory capacities in the capital city. Thus, DENV surveillance mainly focuses on Vientiane Capital. Information collected in the capital city provided fine-tuned data on the balance and dynamics of DENV serotypes during Lao epidemics. Furthermore, as demonstrated previously by Dubos-Pérès et al. [15], and in this study, this system is sensitive enough to catch minor serotypes such as DENV-1. The results also indicated that, even if the majority of the samples tested were collected in Vientiane Capital, the outcome of the surveillance in the capital can be used as a proxy for the situation at the country level. For instance, the surveillance system revealed a parallel situation between Vientiane Capital and most of the rest of the country. DENV-1 represented about 20% of the samples serotyped between 2016 and 2020. Considering the epidemiology of dengue in the Lao PDR, with a succession of outbreaks associated with different DENV serotypes, the risk of an increase in DENV-1 cases cannot be ruled out and deserves further attention in order to predict or even prevent future outbreaks [12,27,28,29,30].

From a genetic viewpoint, even if the number of DENV sequences was limited, the heterogenous geographical and temporal distribution of the samples eligible for the sequencing over the period of study allowed us to obtain an overview of DENV-1 circulation in the Lao PDR. All the DENV-1 strains analyzed here belonged to genotype I. This has been the only DENV-1 genotype described in the Lao PDR up until now [15,16] and in neighboring countries such as China [31], Vietnam [32], Thailand [33], Cambodia [34] and Malaysia [35]. Since 2008, DENV-1 sequences identified in the Lao PDR were distributed within 13 distinct clusters (clusters 1 to 13) of genotype I. They grouped with sequences documented in different countries in Southeast Asia (Thailand, Malaysia, China, Indonesia, Cambodia, Vietnam, Myanmar, Taiwan and Singapore). The newly characterized sequences belonged to clusters 11 and 13 and some of them defined new clusters, i.e., cluster 14 and cluster 15. These new clusters were previously described in 2015 in Taiwan (imported from Myanmar) and between 2017 and 2019 in China, Cambodia and Thailand but were never identified in the Lao PDR before.

These results show that for more than ten years, there has been a rapid turnover of clusters of DENV-1 genotype I isolates due to probable multiple introductions in the Lao PDR from neighboring countries following the trend of this DENV-1 pattern at the regional level [15,16,18,29,36,37,38]. The new genetic data also allows access to a deeper level of analysis of DENV through the clusters’ dynamics. As shown by our results, sequences from samples collected between 2015 and 2018 were distributed in clusters 11 and 13 whereas all but one sequence from the period 2019–2020 fell in clusters 14 and 15. Such switches in clusters or even genotypes, already observed in the Lao PDR for DENV-3 [17] and DENV-2 [19], could have an impact on DENV-1 epidemiology [39,40,41]. This impact of DENV genetic features has recently been described in China where the emergence of a new DENV-1 genotype could be linked to an increase in dengue cases [42]. These dengue epidemiological profile modifications were also observed in New Caledonia for DENV-1 [41] and for other serotypes in India and Malaysia [39,42,43,44]. Therefore, cluster-associated factors, such as the patients’ viremia or vector transmission rates, need to be investigated in the specific context of the Lao PDR to prevent future dengue outbreaks [45]. In the Lao PDR, *Ae. aegypti* is the main arbovirus vector followed by *Ae. albopictus* [18,46,47]. For both species, the abundance of the vectors increases during the rainy season (i.e., mid-April to mid-October), which is often correlated with an increased risk of a dengue outbreak even between the rainy periods [18,48,49]. In the Lao PDR, a vector competence study has previously demonstrated a high transmission rate of DENV-1 by *Ae. aegypti* (transmission efficiency > 50% at 14 days post-infection) [50]. All these data thus emphasize the risk of DENV-1 epidemic re-emergence in the Lao PDR, especially during and after the rainy season.

Geographic specificities of the Lao PDR associated with the development of international population movements and regional trading could influence the emergence and spread of DENV-1 in the country. Interestingly, for more than 10 years, the DENV-1 genotype I was detected in the Lao PDR and at the regional level. Despite the rapid turnover of clusters demonstrated in this study, the long-lasting maintenance of DENV-1 suggests that the Lao population remains susceptible and highly exposed to this serotype. Thus, there is a strong need to develop a combined approach to determine the impact of the host including its immunologic status, vector, virus and their interactions on DENV-1 transmission in the specific context of the Lao PDR to prevent future outbreaks.

## Figures and Tables

**Table 1:** List and position of primers used for RT-PCR and sequencing of the DENV-1 *envelope* gene.

Figure	Forward	Genome Position *	Reverse	Genome Position *
**D1-FG1**	5′CCT-CTG-AAG-GCG-CTT-GGA-A3′	771–789	5′GGC-TCG-TCC-ACA-AAC-AAT-GG3′	1551–1570
**D1-FGT2**	5′AAC-ACC-YCA-AGC-TCC-YAC3′	1426–1443	5′TCT-TGC-ATG-GYG-CRT-CTG3′	1920–1937
**D1-FGT3**	5′TGG-CTG-AGA-CYC-ARC-ATG3′	1869–1886	5′TTG-CTC-TGT-CCA-RGT-GTG3′	2495–2512

* The genome positions are given according to the dengue virus serotype 1 reference genome (GenBank: NC_001477). Primers are described in Balière et al., manuscript in preparation.

**Table 2:** Geographical distribution of dengue virus cases confirmed by the Institut Pasteur du Laos arbovirus surveillance network between 2012 and 2020.

Province	2012	2013	2014	2015	2016	2017	2018	2019	2020	Total
Vientiane Capital	105	467	8	65	286	644	290	870	485	3220
Attapeu	0	0	0	25	6	69	111	62	16	289
Bokeo	0	2	0	0	0	0	0	0	0	2
Bolikhamxay	0	3	0	0	0	0	2	9	13	27
Champasak	0	1	0	0	0	0	3	41	0	45
Huaphanh	0	1	0	0	0	0	0	0	0	1
Khammuane	0	1	0	0	0	3	0	1	0	5
Luangprabang	1	0	0	0	0	0	2	15	18	36
Oudomxay	1	0	0	0	0	0	0	1	1	3
Saravane	0	0	0	0	42	150	29	102	33	356
Savannakhet	0	0	0	0	0	0	19	25	6	50
Vientiane Province	8	38	0	0	0	4	9	37	30	126
Xayaboury	0	1	0	0	0	0	1	2	1	5
Xaysomboun	0	0	0	0	0	0	0	1	4	5
Xiengkhuang	0	0	0	0	0	1	0	12	2	15
Unknown	16	23	3	0	0	0	0	0	0	42
Total	131	537	11	90	334	871	466	1178	609	4227

**Table 3:** Information on Lao DENV-1 isolates.

Scheme	Date of Collection	GenBank Accession Number
LaoPDR, Attapeu, 2015, 3058 *	July 2015	MW559393
LaoPDR, Attapeu, 2015, 3059 *	July 2015	MW559394
LaoPDR, Attapeu, 2015, 3061 *	July 2015	MW559395
LaoPDR, Vientiane Capital, 2016, 4069	July 2016	MW559396
LaoPDR, Vientiane Capital, 2016, 4513	September 2016	MW559397
LaoPDR, Vientiane Capital, 2018, 8225	September 2018	MW559398
LaoPDR, Saravane, 2019, 9393	February 2019	MW559399
LaoPDR, Luangprabang, 2019, 9816	May 2019	MW559400
LaoPDR, Saravane, 2019, 10,167	June 2019	MW559401
LaoPDR, Xayaboury, 2019, 11,062	June 2019	MW559402
LaoPDR, Champasak, 2019, 11,458	June 2019	MW559403
LaoPDR, Vientiane Capital, 2019, 11,672	July 2019	MW559404
LaoPDR, Saravane, 2019, 11,940	July 2019	MW559405
LaoPDR, Attapeu, 2019, 12,069	July 2019	MW559406
LaoPDR, Vientiane Capital, 2019, 12,599	July 2019	MW559407
LaoPDR, Vientiane Province, 2019, 12,922	July 2019	MW559408
LaoPDR, Attapeu, 2019, 13,099	July 2019	MW559409
LaoPDR, Vientiane Capital, 2019, 15,825	December 2019	MW559410
LaoPDR, Saravane, 2020, 15,976	February 2020	MW559411
LaoPDR, Saravane, 2020, 16,094	April 2020	MW559412
LaoPDR, Attapeu, 2020, 16,197	May 2020	MW559413
LaoPDR, Vientiane Capital, 2020, 16,388	July 2020	MW559414
LaoPDR, Vientiane Capital, 2020, 16,396	July 2020	MW559415
LaoPDR, Vientiane Capital, 2020, 17,088	September 2020	MW559416
LaoPDR, Vientiane Capital, 2020, 17,175	September 2020	MW559417
LaoPDR, Vientiane Capital, 2020, 17,256	September 2020	MW559418
LaoPDR, Vientiane Capital, 2020, 17,271	September 2020	MW559419

* Indicates the samples previously sequenced and added to this study for the phylogenetic analysis.

## Data Availability

Not applicable.
